# Breath-hold imaging of the coronary arteries using Quiescent-Interval Slice-Selective (QISS) magnetic resonance angiography: pilot study at 1.5 Tesla and 3 Tesla

**DOI:** 10.1186/s12968-015-0205-2

**Published:** 2015-11-23

**Authors:** Robert R. Edelman, S. Giri, A. Pursnani, M. P. F. Botelho, W. Li, I. Koktzoglou

**Affiliations:** Department of Radiology, NorthShore University HealthSystem, 2650 Ridge Avenue, Evanston, IL 60201 USA; Feinberg School of Medicine, Northwestern University, Chicago, USA; Siemens Medical Solutions USA, Inc., Chicago, USA; The University of Chicago Pritzker School of Medicine, Chicago, USA

**Keywords:** Quiescent-interval slice-selective (QISS), Coronary artery, Magnetic resonance angiography, Breath-holding, Navigator, Free-breathing

## Abstract

**Background:**

Coronary magnetic resonance angiography (MRA) is usually obtained with a free-breathing navigator-gated 3D acquisition. Our aim was to develop an alternative breath-hold approach that would allow the coronary arteries to be evaluated in a much shorter time and without risk of degradation by respiratory motion artifacts. For this purpose, we implemented a breath-hold, non-contrast-enhanced, quiescent-interval slice-selective (QISS) 2D technique. Sequence performance was compared at 1.5 and 3 Tesla using both radial and Cartesian k-space trajectories.

**Methods:**

The left coronary circulation was imaged in six healthy subjects and two patients with coronary artery disease. Breath-hold QISS was compared with T2-prepared 2D balanced steady-state free-precession (bSSFP) and free-breathing, navigator-gated 3D bSSFP.

**Results:**

Approximately 10 2.1-mm thick slices were acquired in a single ~20-s breath-hold using two-shot QISS. QISS contrast-to-noise ratio (CNR) was 1.5-fold higher at 3 Tesla than at 1.5 Tesla. Cartesian QISS provided the best coronary-to-myocardium CNR, whereas radial QISS provided the sharpest coronary images. QISS image quality exceeded that of free-breathing 3D coronary MRA with few artifacts at either field strength. Compared with T2-prepared 2D bSSFP, multi-slice capability was not restricted by the specific absorption rate at 3 Tesla and pericardial fluid signal was better suppressed. In addition to depicting the coronary arteries, QISS could image intra-cardiac structures, pericardium, and the aortic root in arbitrary slice orientations.

**Conclusions:**

Breath-hold QISS is a simple, versatile, and time-efficient method for coronary MRA that provides excellent image quality at both 1.5 and 3 Tesla. Image quality exceeded that of free-breathing, navigator-gated 3D MRA in a much shorter scan time. QISS also allowed rapid multi-slice bright-blood, diastolic phase imaging of the heart, which may have complementary value to multi-phase cine imaging. We conclude that, with further clinical validation, QISS might provide an efficient alternative to commonly used free-breathing coronary MRA techniques.

## Background

Cardiovascular magnetic resonance is an excellent test for evaluating anatomy, function, and blood flow in the heart [1]. It is a valuable adjunct to echocardiography and is routinely used to evaluate a variety of disorders including masses, congenital heart disease, valve abnormalities, inflammatory conditions, and cardiomyopathies. Aside from the recent introduction of quantitative T1 and T2 mapping sequences, cardiac imaging protocols in routine clinical use have remained largely stable over the last decade. Typical imaging protocols consist of a combination of bright blood cine balanced steady-state free precession (bSSFP), 2D phase contrast, dark blood turbo spin-echo, and late gadolinium enhancement scans using inversion recovery-prepared gradient-echo. When coronary artery evaluation is needed, a free-breathing, navigator-gated 3D acquisition is applied [2]. However, a drawback of currently available free-breathing coronary imaging techniques is their dependence on the patient’s respiratory pattern, which can result in inconsistent image quality and unpredictably long scan times.

Quiescent-interval slice-selective (QISS) is a non-contrast-enhanced, bright blood sequential 2D imaging technique that was originally developed for the evaluation of peripheral arterial disease [3, 4]. The original method had temporal resolution on the order of 300 ms, too slow to be applicable for cardiac applications such as coronary MRA. We therefore implemented multi-shot versions of QISS offering higher temporal resolution and compared them with breath-hold T2-prepared 2D bSSFP and standard-of-care free-breathing, navigator-gated 3D bSSFP for imaging of the coronary arteries. In addition, since bSSFP-based imaging techniques can be problematic at high field due to increased specific absorption rate (SAR), B0 and B1 inhomogeneities [5], we also evaluated the performance of these three imaging techniques at both 1.5 Tesla and 3 Tesla.

## Methods

The study was approved by the Institutional Review Board and used written, informed consent. Imaging was performed using a six-element cardiac phased array coil at 1.5 Tesla (MAGNETOM Avanto, Siemens Healthcare, Erlangen, Germany) and 3 Tesla (MAGNETOM Verio, Siemens Healthcare, Erlangen, Germany) with peak gradients and slew rates of 45 mT/m and 200 T/m/s. The prototype QISS pulse sequence was evaluated for imaging of the proximal and mid left coronary circulation of healthy subjects (six male, age range 23–35 years). No pre-medication was administered. In addition, two subjects (both male, ages 39 and 57 years) who had undergone coronary CT angiography for the evaluation of suspected coronary artery disease (CAD) were imaged.

**Fig. 1:**
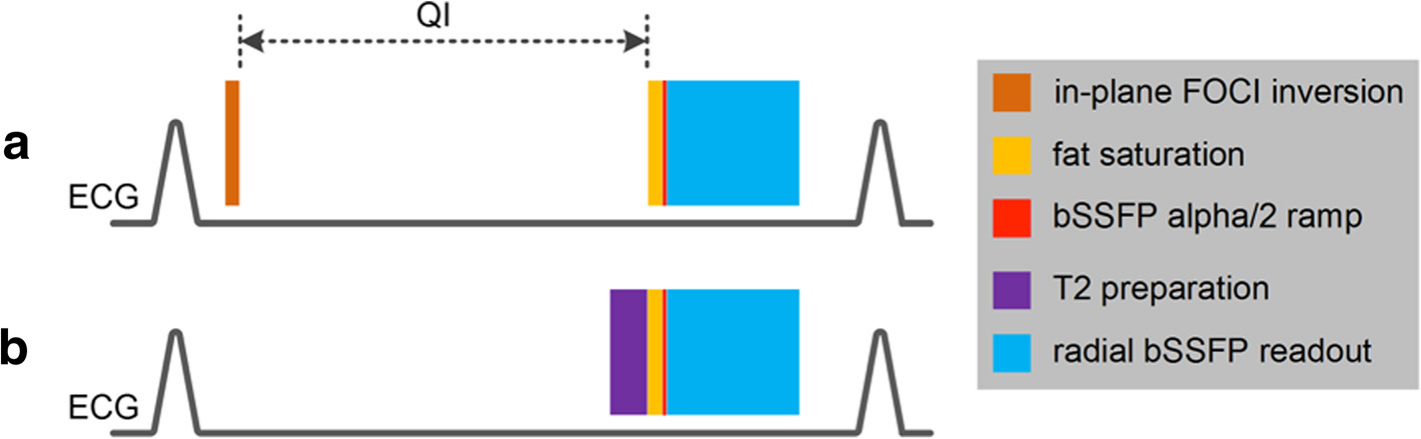
Pulse sequence diagrams for radial QISS (**a**) and 2D T2-prepared bSSFP (**b**). QISS (flow dependent) applies a slice-selective FOCI pulse for inversion of in-plane spins, followed by a quiescent interval (QI) to allow for replenishment of in-plane arterial spins. With T2-prepared bSSFP (flow independent), a spatially non-selective T2 preparation is applied

The differences between the QISS imaging parameters typically used for peripheral artery MRA and coronary MRA are summarized in Table 1. For this pilot study, nearly identical QISS pulse sequence parameters were used for coronary MRA at 1.5 Tesla and 3 Tesla. The pulse sequence diagrams for QISS and 2D T2-prepared bSSFP are given in Fig. 1. An in-plane frequency offset corrected inversion (FOCI) pulse [6] (pulse duration 10.24 ms, μ = 12, β = 900, gradient factor of 2.0) having double the imaging slice thickness was applied immediately after the R-wave to suppress background signal, followed by a quiescent interval to allow inflow of unsaturated arterial spins. Based on empirical experience, the longest quiescent interval that could be accommodated within the RR interval was used. A fat saturation radiofrequency (RF) pulse was applied, followed by an alpha/2 catalyzation, after which data were collected using a 2D bSSFP readout. Sampling bandwidth was 820 or 1008 Hz/pixel resulting in an inter-view repetition time of 4.0 ms or 3.7 ms, respectively.

**Table 1:** Coronary QISS vs. peripheral QISS MRA

	Coronary QISS	Peripheral QISS
K-Space Trajectory	Radial or Cartesian	Cartesian
Readout Duration	~82–192 ms	~300 ms
Magnetization Preparation	FOCI inversion	Saturation
Fat Suppression	Yes	Yes
Venous Suppression	No	Yes
Fold Over Artifact with Small FOV	Cartesian only	Yes

Breath-hold coronal and axial scout images were acquired using single-shot or two-shot radial QISS. Using the coronal scout images to visualize the proximal LAD, single oblique, tilted axial images were obtained for long-axis evaluation of the LAD. The axial scout images were used to center the field of view (FOV) on the heart and, for small FOV radial imaging, to exclude lung tissue in order to ensure an optimal shim (since shimming is only performed on tissue within the selected FOV). For the formal sequence comparisons, breath-hold scans (QISS and T2-prepared bSSFP) were acquired using two shots of 48 views each (total of 96 views), with the shots acquired over two success heartbeats. For Cartesian QISS, the matrix size was 256 × 170, FOV 358-mm × 237-mm, parallel acceleration (ipat) factor of 2. For radial QISS, the matrix was 160 and FOV was 225-mm. A small equidistant azimuthal radial view angle increment ≈ 10–20° was applied since prior experience indicated that this resulted in fewer artifacts than linear or golden angle trajectories [7]. For both k-space trajectories, ten slices were typically acquired in each breath-hold. In-plane spatial resolution was 1.4-mm (0.7-mm after interpolation), with slice thicknesses of 2.1-mm for coronary MRA and 3.1-mm for imaging of the heart.

QISS was compared with a breath-hold T2-prepared 2D bSSFP sequence (TE = 40 ms, identical to QISS except for the magnetization preparation) in order to distinguish the impact of the magnetization preparation from the impact of breath-holding. Additionally, QISS was compared with a free-breathing, navigator-gated fat-suppressed, T2-prepared (TE = 53 ms) 3D bSSFP pulse sequence. For the latter technique, a cross-pair navigator was placed over the right hemi-diaphragm using a ±2.5-mm acceptance window with slice following and adaptive correction; typically 24 slices were acquired using slice thickness, in-plane resolution, and sampling bandwidth identical to those used for QISS.

For quantitative analysis, the contrast-to-noise ratios (CNR) between the coronary arteries and the background tissues of myocardium, epicardial fat and lung were computed as the respective signal differences divided by noise, with noise estimated as the standard deviation of signal within a homogeneous region of the lung tissue located adjacent to the heart. Coronary vessel sharpness was measured as the inverse of the distance between the 20^th^ and 80^th^ percentile points of a signal profile through the left anterior descending artery (LAD) [8]. A fellowship-trained cardiovascular radiologist (MPFB) scored image quality for the left main (LM), LAD and left circumflex (LCx) coronary arteries on a 4-point scale (1: non-diagnostic, 2: poor, 3: good; 4: excellent). Due to the length of time required to run all of the pulse sequence comparisons for the left coronary circulation, additional scan volumes directed to the right coronary artery were not routinely acquired. Consequently, this vessel was not included in the formal analysis.

Differences in quantitative and qualitative scores were assessed using parametric student’s *t* and non-parametric Wilcoxon tests, respectively; statistical tests were paired when comparing matched data. Statistical tests were performed using R software (version 3.2.1, R Foundation for Statistical Computing, Vienna) and *P* values less than 0.05 were considered to indicate significant differences.

## Results and discussion

*Cartesian* vs. *radial QISS:* The mean RR interval was 1020 ms (range 720–1250 ms). QISS image quality was comparable for Cartesian and radial k-space trajectories (P = NS). Coronary sharpness, however, was significantly improved with radial as compared with Cartesian sampling (0.67 ± 0.12 mm^−1^ vs 0.57 ± 0.09 mm^−1^, *P* < 0.01). The improved sharpness may be due to the motion insensitivity of radial k-space trajectories [9]. On the other hand, CNR between the coronaries and background myocardium, pericardial fat, and lung parenchyma was 49 % (37.0 ± 13.0 vs 24.8 ± 9.5), 42 % (50.7 ± 16.0 vs 35.7 ± 11.5) and 47 % (54.2 ± 17.9 vs 36.8 ± 12.4) higher with Cartesian sampling (*P* < 0.001); data expressed as mean ± standard deviation. Radial QISS showed mild streak artifacts due to undersampling. These artifacts were concentrated in the periphery of the image where they did not adversely impact coronary image quality.

**Fig. 2:**
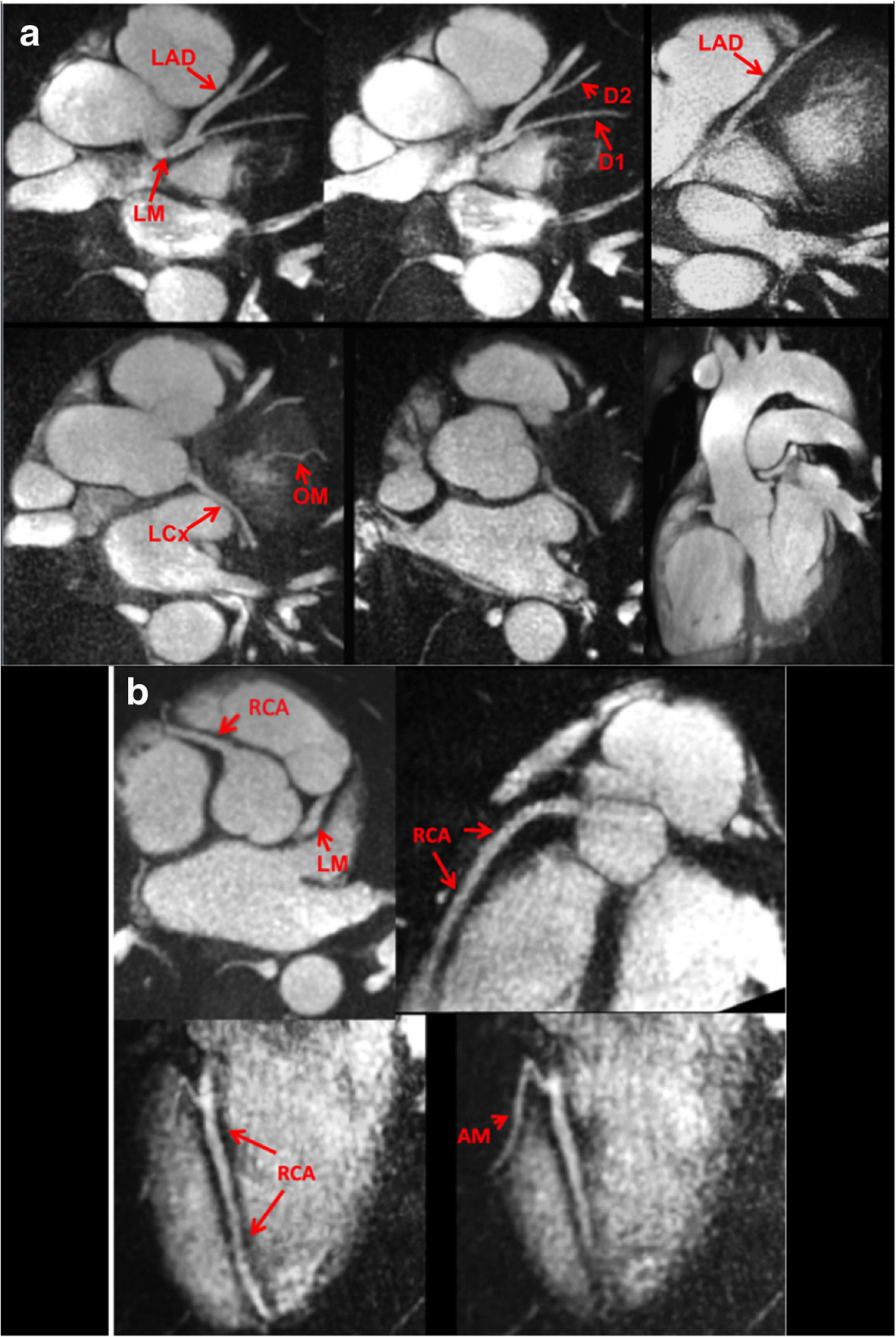
Examples of thin (4 to 10-mm) maximum intensity projections reconstructed from single breath hold, radial QISS (10–12 slices per breath hold, slice thickness = 2.1-mm with 20-50 % slice overlap, in-plane spatial resolution of 0.4-mm to 0.5-mm after interpolation). Images were acquired at 3 Tesla using various scan orientations. **a** Aorta and left coronary circulation. LM = left main; LAD = left anterior descending; D1 = first diagonal branch; D2 = second diagonal branch; LCx = left circumflex; OM = obtuse marginal branch. **b** Right coronary circulation. RCA = right coronary artery; AM = acute marginal branch

**Fig. 3:**
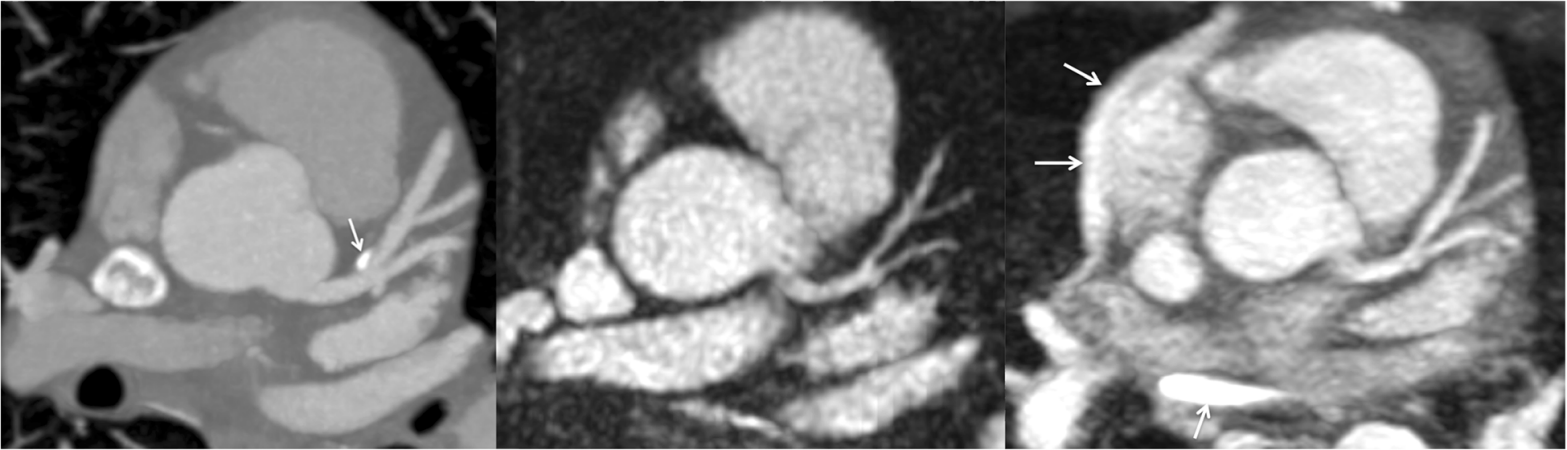
39-year-old male evaluated for chest pain. *Left*: 5-mm MIP of coronary CT angiography shows mild narrowing of the proximal LAD and a punctate coronary calcification (arrow). Middle: MIP of breath-hold radial QISS MRA obtained at 1.5 Tesla demonstrates the left main and LAD coronary arteries, including the D1 and D2 branches. The appearances are similar to the coronary CT angiogram except that the wall calcification is not visible. *Right*: MIP from navigator-gated 3D bSSFP also demonstrates the left coronary anatomy comparably to the coronary CTA. Compared with QISS, there is increased pericardial fluid signal (arrows)

**Fig. 4:**
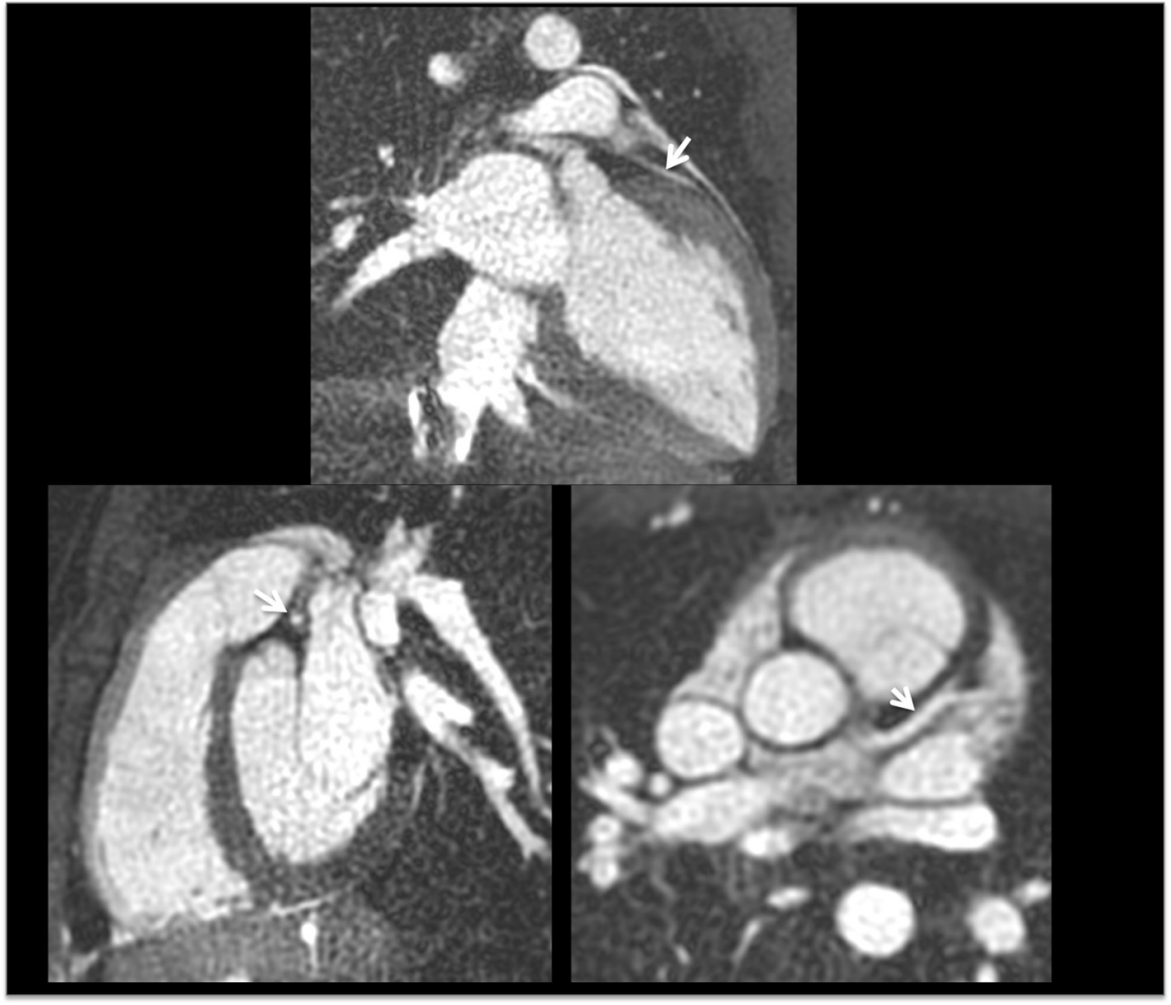
Radial QISS images acquired in three orthogonal planes within a single breath-hold show the LAD (arrow) in long and short axes. Left and right ventricular myocardium, pulmonary veins, and mitral valve leaflets are also well depicted

**Fig. 5:**
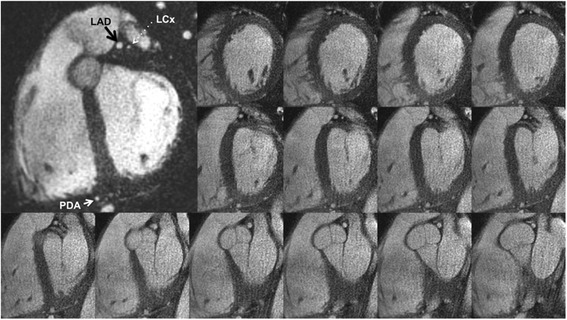
Montage of radial QISS images that were oriented orthogonally to the long axis of the LAD. Images were acquired at 1.5 Tesla in two breath-holds (14 images shown out of 18 acquired). The LAD and LCx, including their takeoffs from the left main coronary artery, are well seen. Magnified view (inset) shows the LAD, left circumflex, and posterior descending branch of the right coronary artery (PDA)

*1.5 Tesla* vs. *3 Tesla:* QISS image quality was excellent at both 1.5 Tesla and 3 Tesla (mean scores of 3.86 and 3.91, respectively, P = NS) (Figs. 2 and 3). QISS images could be acquired in arbitrary planes so as to optimally demonstrate the coronary lumen, either within a single (Fig. 4) or multiple breath-holds (Fig. 5). In addition to the coronary arteries, QISS images provided excellent delineation of the aortic root, ventricular myocardium, pulmonary veins, and pericardium.

Coronary CNR with respect to the myocardium, fat and lung was 1.50-fold (34.1 ± 13.3 vs 22.7 ± 10.2), 1.49-fold (47.6 ± 17.0 vs 32.0 ± 10.9), and 1.50-fold (50.1 ± 19.0 vs 33.4 ± 12.7) higher at 3 Tesla (*P* < 0.001). Stripe artifacts due to off-resonance effects were generally limited to the region of the pulmonary vein ostia and transverse portion of the aortic arch; these artifacts were more prominent at 3 Tesla than at 1.5 Tesla. Coronary sharpness was slightly improved at 3 Tesla versus 1.5 Tesla (0.65 ± 0.12 mm^−1^ vs 0.59 ± 0.10 mm^−1^, *P* < 0.05).

The flip angle for QISS at 1.5 Tesla was fixed at 120°; at 3 Tesla, the flip angle was subject-dependent due to SAR limitations and ranged from 75 to 90°. Whereas QISS involves the application of a single FOCI magnetization preparation pulse, the T2-prepared bSSFP pulse sequence on our scanner applies four adiabatic 180-degree RF pulses during the magnetization preparation, which substantially increases the SAR. At 3 Tesla, due to SAR limitations resulting from the T2-weighted magnetization preparation, both breath-hold T2-prepared 2D bSSFP and navigator-gated 3D bSSFP could only be triggered to every second R-wave, whereas QISS was triggered to every R-wave. Thus, at 3 Tesla only 5 slices could be acquired per breath-hold due to the lower scan efficiency of T2-prepared 2D bSSFP versus 10 slices with QISS.

**Fig. 6:**
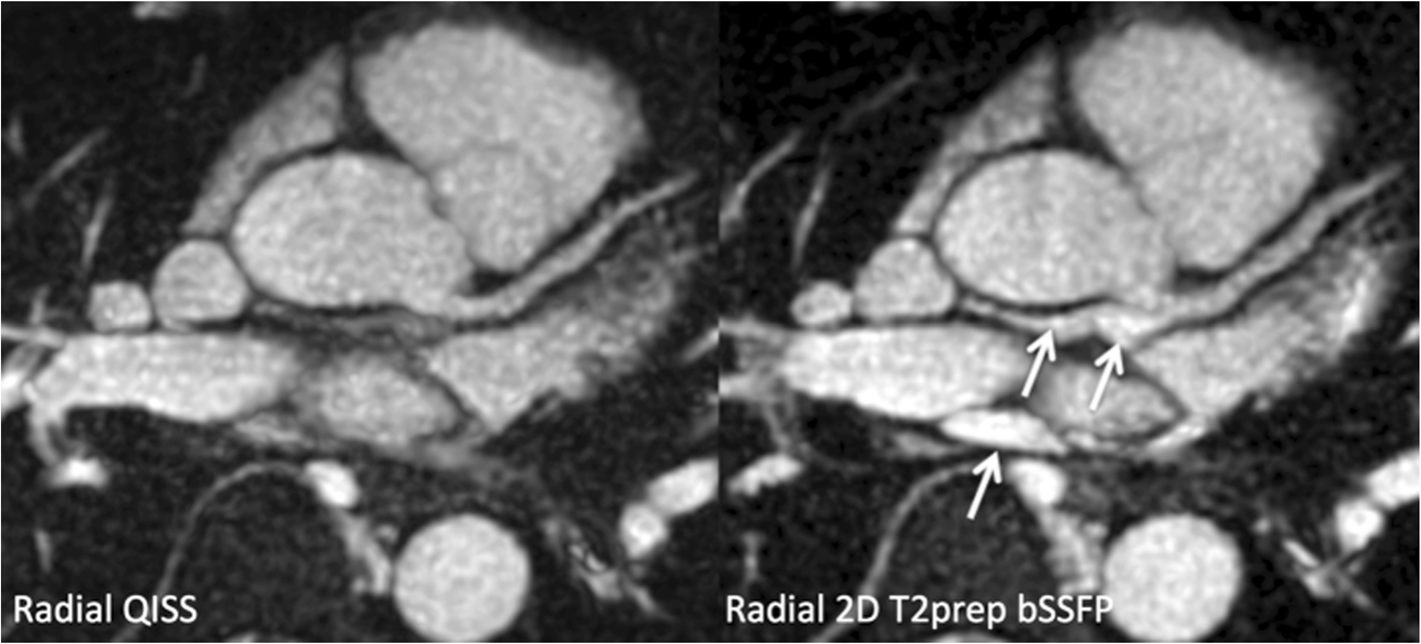
Comparison of source images from breath-hold radial QISS (*left*) and T2-prepared radial 2D bSSFP (*right*) in a healthy volunteer at 1.5 Tesla. Compared with QISS, pericardial fluid signal (arrows) is substantially increased with T2-prepared bSSFP

*Breath-hold QISS* vs. *free-breathing 3D bSSFP:* Scan time for breath-hold QISS was on the order of 20 s for an RR interval of 1 s and 10 slices. Scan time for free-breathing 3D bSSFP ranged from 1 min 57 s to 4 min 3 s at 1.5 Tesla, and 3 min 25 s to 14 min 14 s at 3 Tesla. Breath-hold QISS depicted the left main, LAD including diagonal branches, and the LCx in all subjects without substantial motion artifacts. By comparison, the quality of free-breathing navigator-gated 3D bSSFP was more variable, with image degradation due to respiratory motion in 50 % (6/12) of scans. Fluid in the pericardial recesses appeared brighter on both breath-hold 2D (Fig. 6) and free-breathing 3D T2-prepared bSSFP than with QISS. In no subject did the free-breathing technique outperform QISS with respect to image quality. Averaged over both main magnetic field strengths, mean image quality scores for the left main/LAD/LCx using breath-hold radial QISS and free-breathing navigator-gated 3D coronary MRA were, respectively, 3.91/3.91/3.82 and 3.33/3.46/3.17 (*P* < 0.05 at all three locations). In the LAD, CNR values for radial QISS [navigator-gated coronary MRA] were 26.6 ± 12.0 [26.7 ± 9.83] with respect to myocardium, 36 ± 12.5 [33.7 ± 7.99] with respect to fat, and 37.5 ± 14.4 [39.8 ± 15.0] with respect to lung at 1.5 Tesla. At 3 Tesla, CNR values were 38.8 ± 11.8 [33.5 ± 11.8], 52.4 ± 15.1 [44.4 ± 21.5], and 55.6 ± 17.6 [51.9 ± 20.9] with respect to myocardium, fat and lung, respectively. Compared with free-breathing 3D bSSFP, coronary sharpness was significantly improved with breath-hold coronary QISS (0.62 ± 0.11 mm^−1^ vs 0.43 ± 0.14 mm^−1^, *P* < 0.001).

*Breath-hold QISS* vs. *breath-hold T2-prepared bSSFP:* Coronary sharpness was significantly better with QISS (0.66 ± 0.09 mm^−1^ with QISS vs 0.55 ± 0.12 mm^−1^ with T2-prep bSSFP, *P* < 0.01). QISS image quality was better for the LCx (mean values of 3.98 vs 3.29, *P* < 0.05), but not significantly different for the left main (3.92 vs 3.54) or LAD (4.00 vs 3.73). Coronary-to-myocardium CNR values were 1.6-fold better for QISS than T2-prepared bSSFP (26.8 ± 10.7 vs 16.6 ± 5.2, *P* < 0.05). Corresponding values for coronary-to-fat CNR were 36.3 ± 11.8 vs 25.5 ± 6.4 (*P* < 0.01) and for coronary-to-lung CNR were 37.5 ± 13.2 vs 26.7 ± 6.61 (*P* < 0.01).

**Fig. 7:**
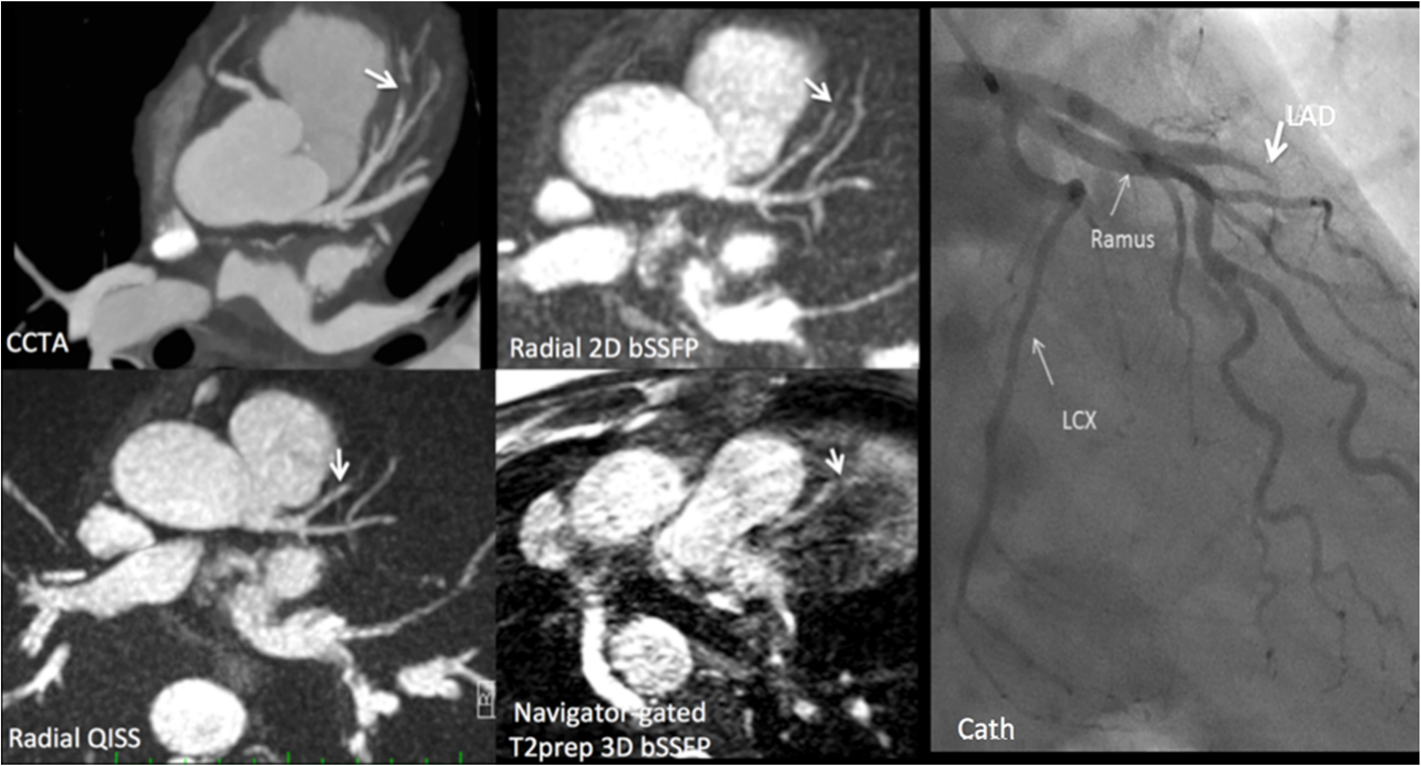
57-year-old patient with hyperlipidemia and chest pain. Breath-hold 2D T2-prepared bSSFP and free-breathing 3D T2-prepared bSSFP showed similar findings to the coronary CTA, which was prospectively interpreted as showing a severe LAD stenosis (arrow). However, radial QISS indicated an LAD occlusion, which was confirmed by subsequent x-ray coronary catheterization

Differences in flow dependence between QISS and T2-prepared bSSFP techniques were anecdotally demonstrated in a patient study (Fig. 7). Breath-hold T2-prepared 2D bSSFP and navigator-gated T2-prepared 3D bSSFP, both of which are substantially flow-independent, suggested a severe LAD stenosis as was prospectively reported on the coronary CTA. However, radial QISS, which is flow-dependent, showed a vessel cut-off indicating an LAD occlusion that was confirmed on subsequent x-ray coronary catheterization.

For more than two decades, attempts have been made to use breath-hold 2D and 3D MRA techniques to image the coronary arteries [10–13]. To date, suboptimal image quality (e.g., due to low spatial resolution, poor flow contrast and/or motion artifact) along with a lack of robustness has impeded the widespread adoption of breath-hold coronary MRA techniques into clinical practice. This pilot study demonstrated that multi-shot QISS MRA has the potential to overcome many of the limitations of previously described breath-hold MRA techniques, enabling consistent, artifact-free imaging of the coronary arteries at both 1.5 Tesla and 3 Tesla.

The QISS technique, as originally described for peripheral nonenhanced MRA, is a flow-dependent, cardiac-gated 2D single-shot acquisition. Arterial flow contrast is maximized by the combination of in-plane and fat saturation RF pulses to suppress signal from stationary spins, along with a quiescent interval of a few hundred milliseconds in order to allow full replenishment of saturated in-plane arterial spins. For coronary MRA, the temporal resolution of the bSSFP readout needs to be improved, which is accomplished by using a multi-shot acquisition. Moreover, the quiescent interval is substantially lengthened, which maximizes replenishment of in-plane arterial spins when the coronary artery is viewed in long axis. Given the lengthy quiescent interval, optimal suppression of myocardial signal is obtained using in-plane inversion with a FOCI RF pulse instead of the saturation pulse typically used for peripheral MRA.

Coronary QISS bears some similarities to flow-dependent inversion-prepared 3D MRA techniques in widespread use for renal MRA [14]. However, there are substantial differences as well. For instance, free-breathing renal MRA requires respiratory gating. Although breath holding is theoretically possible with a 3D acquisition, efficiency is limited by the non-rectangular slab profile when small numbers of slices are acquired. Compared with QISS, flow contrast is inferior since there is much less replenishment of saturated arterial spins between sequence repetitions using a thick-slab 3D acquisition compared with a thin-slice 2D QISS acquisition. It should also be noted that, unlike the case of a thick-slab 3D acquisition, the sequential acquisition of thin 2D slices with QISS causes negligible signal saturation of the aorta and cardiac chambers irrespective of slice orientation.

In comparing Cartesian and radial k-space trajectories for QISS, we found that CNR was better with Cartesian QISS while coronary sharpness was better with radial QISS. From theory, it can be predicted that radial images should have about 87 % of the signal-to-noise ratio of a corresponding Cartesian image [15]. Nonetheless, we found that a radial k-space trajectory has several benefits that make it the preferred approach. Compared with Cartesian, radial k-space trajectories are less sensitive to motion artifacts and provide more flexibility in trading off spatial and temporal resolution, which is helpful for coronary MRA given the range of vessel sizes and heart rates that may be encountered. Radial QISS is immune from fold over artifacts, which allows the use of much smaller FOV than is practical using Cartesian imaging. With radial QISS, the high degree of background suppression from the combination of in-plane tissue inversion and fat suppression minimizes streak artifacts, which facilitates the use of high undersampling factors.

QISS is a flow-dependent MRA technique whereas T2-prepared bSSFP techniques are substantially flow-independent. This difference is anecdotally illustrated by Fig. 7 in which QISS accurately depicted an LAD occlusion, whereas flow-independent techniques (T2-prepared 2D and 3D bSSFP as well as coronary CTA) incorrectly suggested a severe stenosis due to retrograde filling of the distal LAD segment from collaterals. However, our clinical experience with CAD is very limited. In some circumstances the flow dependence of QISS might prove to be a limitation, in which case the additional acquisition of a flow-independent T2-prepared 2D bSSFP MRA could be helpful.

Free-breathing T2-prepared 3D bSSFP is the current mainstay for coronary MRA [16]. However, in our study, SAR limitations at 3 Tesla arising from the four adiabatic 180° RF pulses in the T2-weighted magnetization preparation necessitated triggering to every second R-wave, whereas SAR was not a significant impediment for QISS. Another limitation of the T2-prepared approaches was bright signal from pericardial fluid. In some subjects, high signal from fluid within the inferior aortic recess [17] obscured the proximal portion of the left main coronary artery in thin MIPs, which did not occur with QISS. Alternatively, one could use a low SAR, inversion-prepared 3D spoiled gradient-echo technique [18]. However, unlike non-contrast-enhanced QISS, it requires the slow infusion of a relatively high dose of gadolinium-based contrast agent.

The performance of commercially available free-breathing techniques is predicated on the patient’s respiratory pattern. Consequently, image quality can be unpredictably degraded and scan times inordinately lengthened when the respiratory pattern is irregular [19]. By comparison, image quality with QISS should be consistent so long as the subject is able to sustain a breath hold. Breath-hold times can be reduced as needed, although at the expense of multi-slice capability. The combination of short scan times and immediate image reconstruction should be helpful in providing rapid feedback about image quality and parameter optimization, as well as in determining the need for additional scan planes.

Whole-heart coverage is achievable with free-breathing 3D coronary MRA [20]. By comparison, a drawback of QISS is that it provides substantially less volume coverage in each breath-hold scan. For instance, a QISS scan with ten 2.1-mm thick contiguous, non-overlapping slices only spans a 21-mm thick volume. Nonetheless, QISS allows extensive lengths of a coronary artery to be imaged in each single breath-hold. In our study, coronary arteries were well depicted in all subjects including routine visualization of small LAD branch vessels. Another drawback of QISS compared with free-breathing 3D techniques is the inability to image with isotropic spatial resolution. However, slices can be overlapped as needed to improve the delineation of particular vessel segments or to enhance the quality of 3D multi-planar reformats. A potential advantage of QISS compared with free-breathing techniques may be its ability to simultaneously image a coronary stenosis in both long-axis and short-axis views within a single breath-hold. Moreover, the spatial resolution of a cross-sectional QISS image can be increased as desired to better evaluate the severity of a stenosis. Signal averaging can be performed to compensate for signal-to-noise loss with the smaller pixel, although at the expense of multi-slice capability. While theoretically possible to image a coronary artery in multiple slice orientations and with arbitrary spatial resolutions using free-breathing 3D techniques, scan time constraints make it impractical to do so in clinical practice.

Despite the much shorter scan time of QISS, coronary CNR values were comparable between free-breathing 3D bSSFP and breath-hold QISS scans at 1.5 Tesla. Although the lengthier free-breathing scan benefits from the intrinsic signal averaging of a 3D acquisition, this benefit may be offset by the much greater inflow of unsaturated spins with the 2D acquisition and elimination of noise from respiratory motion artifact by breath-holding [21]. At 3 Tesla, coronary CNR was significantly better for QISS than free-breathing 3D, which may in part be due to the SAR-dependent decrease in efficiency for the latter technique. Respiratory motion artifacts degraded coronary artery image quality in half the free-breathing 3D bSSFP scans but none of the breath-hold QISS scans. In no subject did a free-breathing scan outperform breath-hold QISS. However, our results may not be generalizable to other MRI systems, since free-breathing 3D techniques are highly system and vendor dependent. More advanced approaches under development, such as continuous scanning golden angle MRA [22], will likely provide more consistent image quality with reduced scan time.

The QISS data acquisition was restricted to the diastolic phase of the cardiac cycle for this study. This was done in order to minimize cardiac motion and to ensure that the in-plane FOCI pulse (applied immediately after the R-wave) was coincident with the imaging slice at the time of data acquisition. Since the FOCI pulse has twice the thickness of the imaging slice, image contrast should not be altered if the imaging slice moves by just a few millimeters. However, for a systolic acquisition the myocardium will move to a substantially different position than the one to which the FOCI pulse was applied, which would result in increased background signal. This limitation is not encountered when a spatially non-selective T2-weighted magnetization preparation is used.

Further improvements in QISS image quality should be readily achievable. Our studies were performed on older generation 1.5 Tesla and 3 Tesla MRI systems. Newer generation systems provide a substantial boost in SNR (up to 50 %) through improved phased array coil designs and RF electronics. Compressed sensing techniques have the potential to greatly improve QISS image quality, particularly given the high degree of background signal suppression and resultant sparsity [23]. Multi-slice imaging efficiency can also be improved. It may be possible to at least double the number of slices per breath-hold through the use of simultaneous multi-slice imaging [24].

Temporal resolution is another important consideration, since the coronary arteries will appear blurred if temporal resolution is insufficient. For subjects with rapid heart rates, one can enhance temporal resolution by using fewer radial views or by acquiring more shots. Although the use of more shots in conjunction with a faster heart rate increases scan time, this effect is largely compensated by the proportionately shorter RR interval. An alternative approach might be to use a golden angle radial trajectory. One could then reconstruct QISS images from subsets of data providing arbitrarily high temporal resolution in order to minimize blurring from coronary motion [25]. Additionally, beta-blockers can be administered to slow the heart rate, as is routinely done for coronary CT angiography.

Aside from coronary MRA, QISS may have complementary value to cine bSSFP for evaluating cardiac morphology. While an excellent technique for measuring ventricular function, multi-phase cine bSSFP is inefficient for anatomic evaluation since only a few cine slices can be acquired in each breath-hold. By comparison, single-phase QISS allows the entire left ventricle to be imaged in just a few breath-holds. Unlike cine bSSFP, QISS permits the use of fat saturation to improve contrast between epicardial fat and the myocardium and coronary arteries. Other potential clinical applications include evaluation of the aortic root, pulmonary veins, intra-cardiac masses and pericardial disease.

A limitation of this pilot study is that imaging was performed in cooperative subjects. Free-breathing techniques will likely prove advantageous in sicker patients who are unable to breath-hold. Our study design involved comparisons of multiple pulse sequences, which did not allow time for formal evaluation of the right coronary artery. Parameter modifications, such as the use of more shots for higher temporal resolution and optimization of the quiescent interval so that data are collected during the period of least coronary artery motion, might be beneficial for imaging of the right coronary artery due to its greater mobility compared with the left coronary circulation. Another concern for QISS is that diaphragm drift may cause subtle changes in position for slices acquired late in the breath-hold compared with those acquired earlier. Potential solutions include shorter breath-holds, as well as prospective navigator-based slice correction [26].

## Conclusions

Breath-hold QISS MRA provides a simple, versatile, and time-efficient alternative to free-breathing 3D techniques for evaluation of the coronary arteries at both 1.5 Tesla and 3 Tesla. Initial results suggest advantages for radial QISS, including improved image sharpness, relative insensitivity to flow and motion artifacts, and absence of fold over artifact with small FOV. The consistently short scan times and ease of use should facilitate incorporation into routine cardiac imaging protocols. Future efforts will be directed towards clinical validation in patients with CAD and other cardiovascular disorders.

## Abbreviations

QISS: Quiescent-interval slice-selective
MRA: Magnetic resonance Angiography
LAD: Left anterior descending coronary artery
LCx: Left circumflex coronary artery
LM: Left main coronary artery
RCA: Right coronary artery
SNR: Signal-to-noise ratio
CNR: Contrast-to-noise ratio
bSSFP: Balanced steady-state free precession
RF: Radiofrequency
SAR: Specific absorption rate
FOCI: Frequency offset corrected inversion
